# Genetic diversity and population structure of an African yam bean (*Sphenostylisstenocarpa*) collection from IITA GenBank

**DOI:** 10.1038/s41598-022-08271-4

**Published:** 2022-03-15

**Authors:** Ndenum Suzzy Shitta, Nnanna Unachukwu, Alex Chukwudi Edemodu, Abush Tesfaye Abebe, Happiness O. Oselebe, Wosene Gebreselassie Abtew

**Affiliations:** 1 Jimma University, Jimma, Ethiopia; 2 Ebonyi State University, Abakaliki, Nigeria; 3 International Institute of Tropical Agriculture, Ibadan, Nigeria

**Keywords:** Molecular biology, Plant sciences

## Abstract

African yam bean, AYB (*Sphenostylis* *stenocarpa*), is an underutilized legume of tropical Africa. AYB can boost food and nutritional security in sub-Saharan Africa through its nutrient-rich seeds and tubers. However, inadequate information on germplasm with desirable agro-morphological traits, including insufficient data at the genomic level, has prevented the full exploitation of its food and breeding potentials. Notably, assessing the genetic diversity and population structure in a species is a prerequisite for improvement and eventual successful exploitation. The present study evaluated the population structure and genetic diversity of 169 accessions from the International Institute of Tropical Agriculture (IITA) collection using 26 phenotypic characters and 1789 single nucleotide polymorphism (SNP) markers. The phenotypic traits and SNP markers revealed their usefulness in uniquely distinguishing each AYB accession. The hierarchical cluster of phenotypes grouped accessions into three sub-populations; SNPs analysis also clustered the accessions into three sub-populations. The genetic differentiation (*F*_*ST*_) among the three sub-populations was sufficiently high (0.14–0.39) and significant at *P* = 0.001. The combined analysis revealed three sub-populations; accessions in sub-population 1 were high yielding, members in sub-population 2 showed high polymorphic loci and heterozygosity. This study provides essential information for the breeding and genetic improvement of AYB.

## Introduction

African yam bean (*Sphenostylis* *stenocarpa* Hochst ex. A. Rich. Harms), otherwise known as “AYB,” is a nutritionally rich seed and tuber producing crop of tropical Africa. AYB is a diploid species with a chromosome count of 2n = 22^1,2^. Its flowers are cleistogamous and mainly exhibit self-pollination (91%)^3^. AYB genome size is presently unknown. AYB seeds contain sufficient protein, approximately 37%, and about 64% carbohydrates^4,5^. The protein content in tubers is about 16%, and its carbohydrate content is roughly 68%^6^.

Consequently, the seeds, tubers, and leaves are extensively used in various dietary preparations; the seeds could be roasted, boiled, used as a spice, or processed into a paste^7,8^. Furthermore, the fresh tubers are usually roasted or boiled; the leaves are also boiled and utilized as vegetables^9^. Categorized as an underutilized legume^10,11^, AYB is mainly cultivated among smallholder farmers; these farmers also play significant roles in maintaining the crop’s genetic resources^10,11^. In addition, IITA GenBank presently conserves about 200 accessions. However, farmers’ interest in cultivating the crop is seen as dwindling^10,12^; the diminishing interest could be linked to the identified limitations. Significant among them are; long cooking hours of about 6–24 h, the abundance of anti-nutritional factors in seeds, and an extended maturity cycle of about 9–10 months^13,14^. Realizing the enormous potential of AYB and the constraints associated with the crop, there is a need to investigate variations across the species. The variations identified can be explored to develop improved or entirely new varieties^15^. Characterization at the phenotypic and genotypic levels is reportedly the most widely used in accessing variations in plant studies^15,16^. The phenotypic approach has wide applications in detecting unique characters, identifying duplicates, and selecting germplasm with desirable features^17,18^. Nevertheless, the environment could influence phenotypic studies^19^; because it’s dependent on visual identification, it could be subjective and time-consuming^20^.

In contrast, genotypic characterization is selectively neutral and not affected by the environment^21^. Presently, advances in DNA technology have resulted in high-throughput sequencing approaches facilitating single nucleotide polymorphisms (SNPs) identification and its application in crop improvement. Diversity Array Technology (DArT) is a good example of high throughput technology characterized by a relatively low cost, high call rates, and high reproducibility. DArT is, most importantly, capable of generating genome-wide SNPs in species with no prior DNA sequence information Edet et al.^22^ and Barilli et al.^23^. such as AYB. The DArT approach has proved helpful in genetic studies of several legumes, including; pigeon peas^24^, common beans^25^, and soybeans^26^. Notably, the science of plant breeding centers on identifying and utilizing genetic variation^21^, tracking potential DNA markers and regions associated with traits of interest^27,28^. However, as no single characterization approach is superior to the other^29^ and no method has been identified to be sufficient for evaluating every aspect of a species^30^, characterization based on phenotypic traits and genotypic data can be used both independently or to complement one another^31^.

Therefore, characterizing AYB using a multifaceted approach is a step in the right direction. Previously, authors used phenotypic traits to assess the diversity of about 100 accessions evaluated in Nigeria^17,32^. Additionally, few studies reported PCR-based markers for genetic evaluation across selected accessions^33–35^. A preliminary study using the DArTseq approach in identifying a few SNPs in AYB was recently reported^36^. However, no report is available on high throughput sequencing data for genetic diversity and population structure analysis in the crop.

Similarly, not enough attempt has been made to phenotypically characterize large AYB accessions outside Nigeria and evaluate the genetic diversity and population structure using SNPs. Also, information is lacking on combined analysis at the phenotypic and genotypic levels using a high throughput approach. Moreover, the scanty information presently available at the phenotypic and genotypic level of the crop needs to be complemented with extensive data set, which would increase the probability of finding a genetic correlation between SNPs and phenotype^37^. Therefore, considering the above, the objectives of this study were to evaluate the population structure, genetic diversity, and the differentiation between phenotypic and genotypic data of 169 AYB accessions obtained from IITA’s Genbank collection.

## Results

### Phenotypic differentiation and diversity across 169 AYB accessions

The principal component analysis (PCA) revealed the most discriminative phenotypic traits across the 169 accessions. The traits that largely contributed to the observed variation in each PC axis are shown in bold (Table 1). Days to 1st flowering, days to 50% flowering, dry seed matter, petiole length, seed moisture content (SDMC), terminal leaf length (TLL), terminal leaf width (TLW), 100 seed weights, and seed color are traits that had high loading on more than one principal components (PC). The first eight PCs cumulatively explained 68.68% of the total phenotypic variation; the eigenvalues of the eight PCs varied from 1.11 to 4.81. PC1 made the highest contribution of 18.48% of the total variations, and nine quantitative traits contributed most in the PC axis. PC2 accounted for 13.63% of the total variation of which two quantitative traits; dry seed matter, seed moisture content, and four qualitative traits; mainstem pigmentation (MASPIG), branch pigmentation (BRAPIG), petiole pigmentation (PETPIG), and seed color, contributed most to the observed variation. The traits, dry seed matter, petiole lenght, terminal leaf length, and terminal leaf width, were the main traits that contributed to the observed variation in PC3. PC4 accounted for 7.42% of the total variation across the accessions. The traits that contributed most to the observed variations in PC4 were days to 1st flowering, days to 50% flowering, and 100 seed weight. Seed thickness, 100 seed weight (PC5); seed variegation, seed color (PC6); flower color (PC7), and growth habit (PC8) contributed 5.93%, 5.40%, 5.05%, and 4.25%, respectively, to the total variations across the accessions.

**Table 1:** Principal components and phenotypic traits contribution on each factor.

Phenotypic traits	PC1	PC2	PC3	PC4	PC5	PC6	PC7	PC8
Days to 1st flowering	**0.22**	0.01	0.02	**0.34**	0.04	0.02	0	0.04
Days to 50% flowering	**0.4**	0.01	0.03	**0.25**	0.03	0	0	0
Days to germination	**0.26**	0	0.06	0.14	0	0.04	0	0
Dry seed matter (%)	0.19	**0.22**	**0.35**	0.05	0.08	0.01	0.01	0
Number of seeds per pod	**0.25**	0.07	0.02	0.01	0.04	0.02	0	0.01
Pod length (cm)	**0.39**	0.01	0.07	0.03	0.04	0.02	0.14	0
Petiole length (cm)	**0.3**	0	**0.2**	0.01	0.09	0.1	0.1	0
Seed length (mm)	0.09	0.03	0.11	0.12	0.01	0.01	0.16	0
SDMC (%)	0.19	**0.22**	**0.35**	0.05	0.08	0.01	0.01	0
Seed thickness (mm)	0.07	0.05	0.04	0.15	**0.24**	0.01	0.1	0.09
Seed width (mm)	0.01	0.04	0.05	0.15	0	0	0.06	0.16
Total germination	0.18	0.07	0	0.18	0.03	0.1	0.02	0.01
TLL	**0.37**	0	**0.24**	0	0.1	0.05	0.01	0
TLW	**0.4**	0.01	**0.22**	0	0.03	0.09	0.03	0
Total seed weight (g)	**0.34**	0.19	0.06	0.01	0.01	0	0.01	0.02
100 seed weight (g)	0.05	0.04	0.03	**0.23**	**0.33**	0.01	0.02	0
MASPIG	0.18	**0.63**	0.02	0.05	0.01	0.08	0.01	0
BRAPIG	0.17	**0.62**	0.02	0.05	0.01	0.09	0.01	0
PETPIG	0.18	**0.63**	0.02	0.05	0.01	0.08	0.01	0
Flower colour	0.02	0.02	0.05	0	0.01	0.03	**0.37**	0.04
Growth habit	0.04	0.08	0	0	0.14	0.01	0.01	**0.35**
Pod morphology	0.18	0	0	0.02	0	0	0.13	0
Pod shattering	0.06	0	0.01	0.01	0.09	0.02	0.07	**0.28**
Seed variegation	0.12	0.19	0.11	0.03	0.06	**0.25**	0	0.01
Seed colour	0.04	**0.27**	0.11	0.01	0.01	**0.29**	0.02	0.02
Seed shape	0.08	0.12	0	0	0.05	0.07	0	0.04
Eigenvalue	4.81	3.54	2.21	1.93	1.54	1.40	1.31	1.11
Proportion	18.48	13.63	8.51	7.42	5.93	5.40	5.05	4.25
Cumulative	18.48	32.11	40.63	48.05	53.98	59.37	64.43	68.68

*PC* principal component, *SDMC* seed moisture content, *TLL* terminal leaf length, *TLW* terminal leaf width, *MASPIG* main stem pigmentation, *BRAPIG* branch pigmentation, *PETPIG* petiole pigmentation.

Values in bold (0.2) represent traits that contributed majorly to the observed variations in each PC axis.

**Figure 1:**
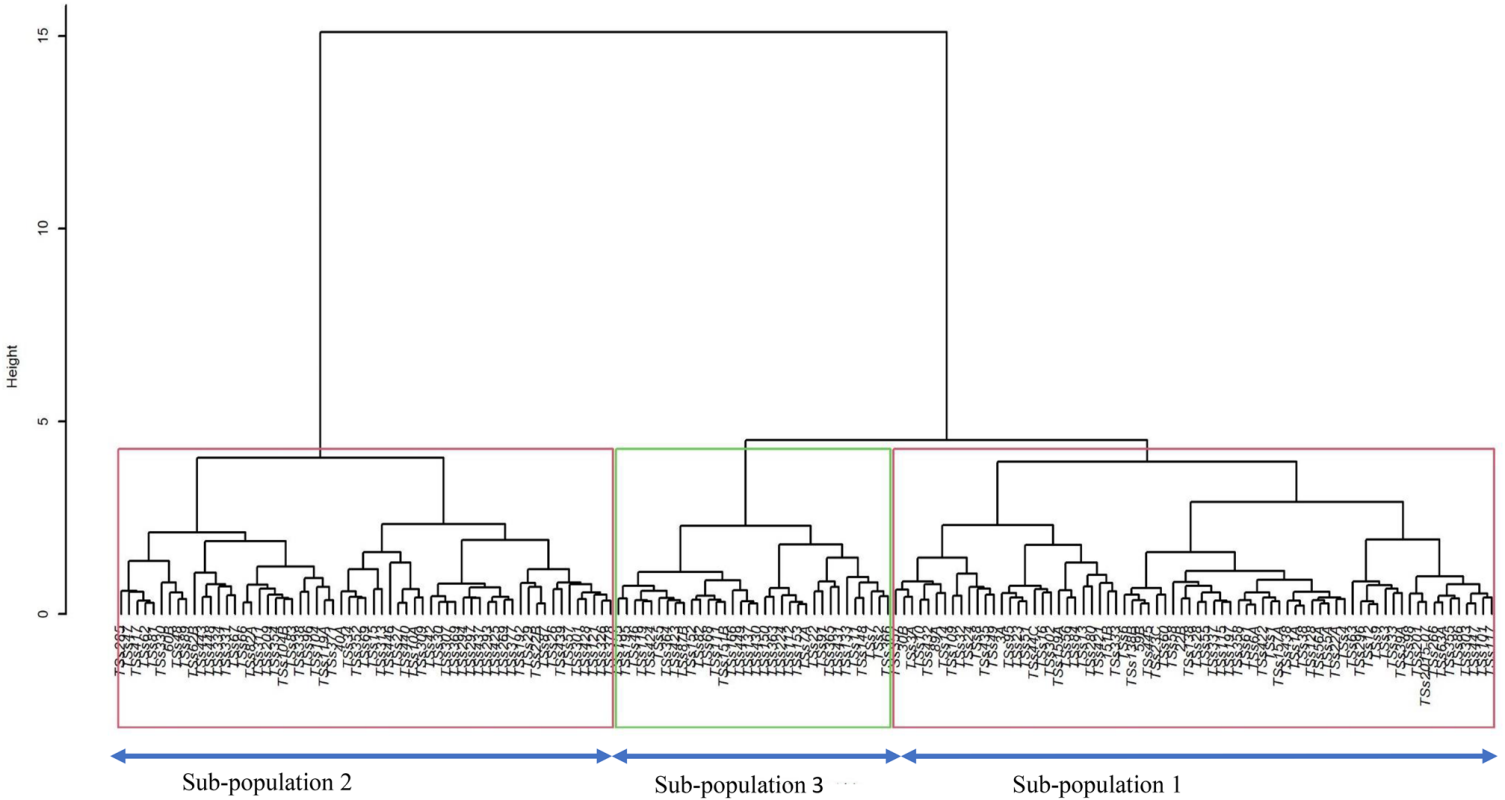
Hierarchical cluster dendrogram based on Gower distance matrix of 26 phenotypic traits. The dendrogram was created in R software version 4.1.1^56^ based on Ward.D2 clustering method.

The genetic distance among the accessions based on their phenotypic evaluation varied from 0.06 to 0.57, with an average of 0.27. The maximum distance was observed between TSs-363 and TSs-446, whereas the minimum distance was observed between TSs-445 and 59B. Furthermore, the hierarchical cluster dendrogram grouped the 169 accessions into three major clusters representing three sub-populations (Fig. 1). Sub-population 1 had the highest number of accessions (72), followed by sub-population 2 (61) and sub-population 3 (36) accessions. The goodness of fit of the cluster dendrogram showed a high cophenetic correlation coefficient of 0.89. The mean values (Supplementary Table S3) of the sub-population showed that accessions grouped in sub-population 1 produced more grain (66.93 g) and were significantly different from sub-population 3 (53.06). More so, the number of seeds per pod (12.18), pod length (16.77 cm), and seed moisture content (7.00%) of accessions in sub-population 1 were higher and significantly different than observed in sub-population 3. Sub-population 3 presented a pod length of 15.77 cm, seed moisture content of 6.58%, and number of seeds per pod of 11.50. About 54% of accessions in sub-population 1 were none pod-shattering; the sub-population was different from sub-population 3.

The correlation among the 10 qualitative traits (Supplementary Fig. S1) showed a positive correlation for all the qualitative traits evaluated. A strong correlation (0.95) in the forward and backward direction was observed between main stem pigmentation (MASPIG) and branch pigmentation (BRAPIG). Likewise, a moderate correlation (0.55) was obtained between seed variegation (SEDVAR) and seed color (SEDCOL) in the backward direction. Furthermore, the correlation among the 16 quantitative traits (Supplementary Fig. S2) showed a statistically significant correlation at P < 0.001 for most of the quantitative traits evaluated. Seed moisture content (SDMC) and dry seed matter (DRMAT) showed highly significant (P < 0.001) and perfect negative correlation (-1.00). Highly significant (P < 0.001) and strong correlation (0.67) was observed between days to 1st flowering (DISFL) and days to 50% flowering. More so, a highly significant, moderate, and positive correlation (0.58) was observed between total seed weight (TSDWT) and seed moisture content; however, a negative (−0.58) but highly significant correlation was found between total seed weight and dry seed matter.

### Genetic diversity and population structure of AYB accessions

**Figure 2:**
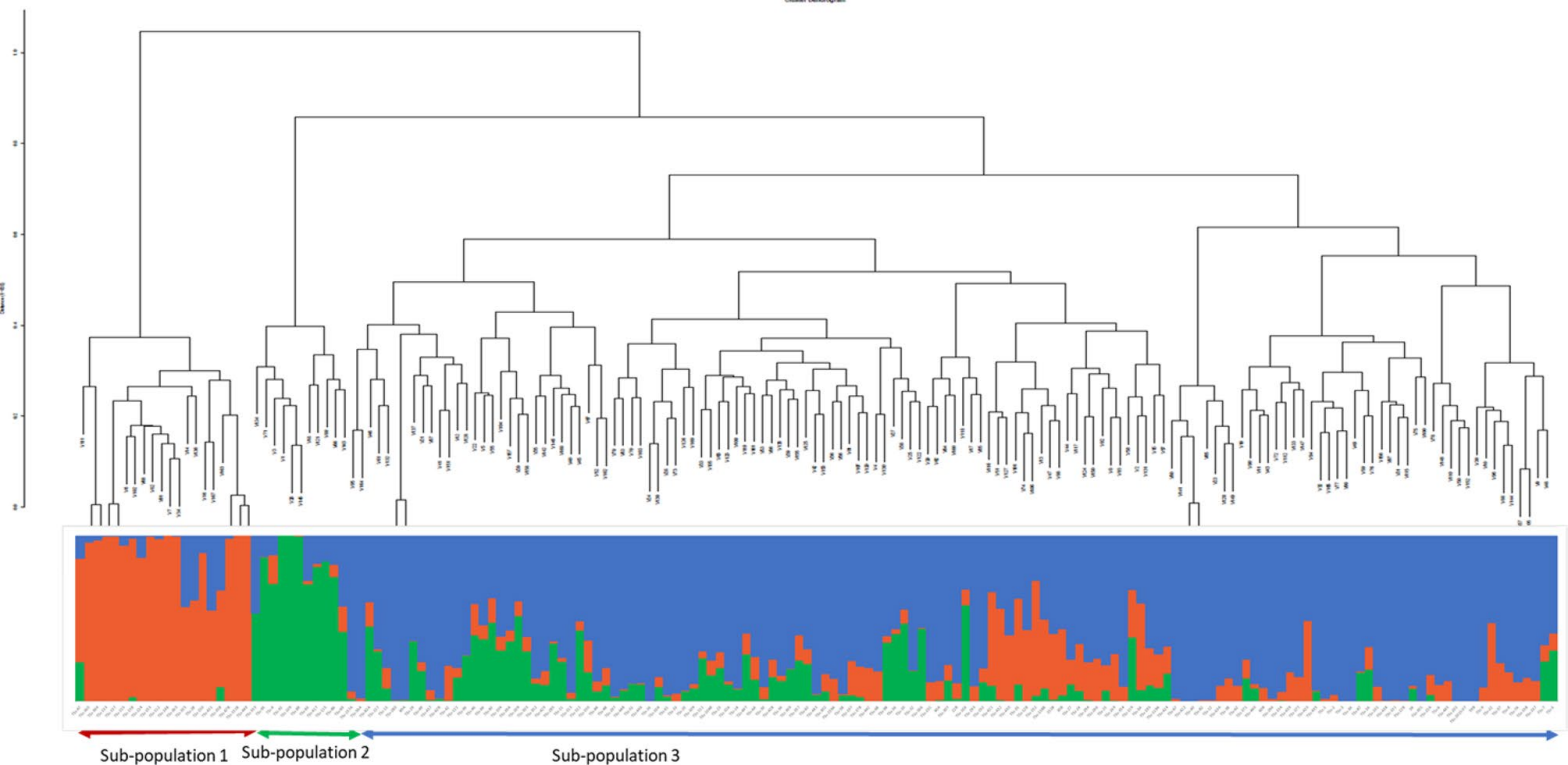
Hierarchical cluster dendrogram showing the genetic relationship of 169 AYB accessions detected with 1789 SNP markers and a population structure plot showing the grouping of the accessions into three sub-populations. Each vertical bar represents an accession. The sub-populations identified are; sub-population 1 (orange), sub-population 2 (green) and sub-population 3 (blue). The dendrogram was created in R software version 4.1.1^56^.

A total of 1789 SNPs from DArTseq was used in studying the genetic diversity of 169 AYB germplasm of IITA collections. The number of effective alleles (Ne) in the population was 1.61, and Shannon’s information index (I) was 0.59. The population’s expected heterozygosity (He) and observed heterozygosity (Ho) were 0.35 and 0.15, respectively. Across the 1789 SNPs, the minor allele frequency ranged from 0.05 to 0.5 with an average of 0.22, and the major allele frequency ranged from 0.50 to 0.95 with an average of 0.78 (Table 2). The genetic distance of the studied accessions based on the Identity-By-State dissimilarity matrix varied from 0.004 to 0.41, with an average of 0.29. The maximum distance (0.41) was observed between accessions TSs-109 and TSs-23C, whereas the minimum (0.004) distance was obtained between TSs-151B and TSs-449. The cophenetic coefficient correlation of the dissimilarity matrix was 0.73, confirming the accuracy of the matrix used for cluster generation. The constructed hierarchical cluster dendrogram separated the accessions into three major clusters representing three sub-populations (1, 2 and 3) (Fig. 2). Sub-population 3 had the highest number of accessions (138), followed by sub-populations 1 (20), and sub-population 2 had the least number of accessions (11).

**Table 2:** Mean allelic patterns across 169 AYB accession.

Parameters	Mean values ± SE
Ne	1.61 ± 0.008
I	0.59 ± 0.004
He	0.35 ± 0.003
Ho	0.15 ± 0.002
Minor allele frequency	0.22 (0.05–0.50)
Major allele frequency	0.78 (0.50–0.95)

*SE* standard error, *Ne* number of effective alleles, *I* Shannon’s information index; number of private alleles, *He* expected heterozygosity, *Ho* observed heterozygosity.

**Figure 3:**
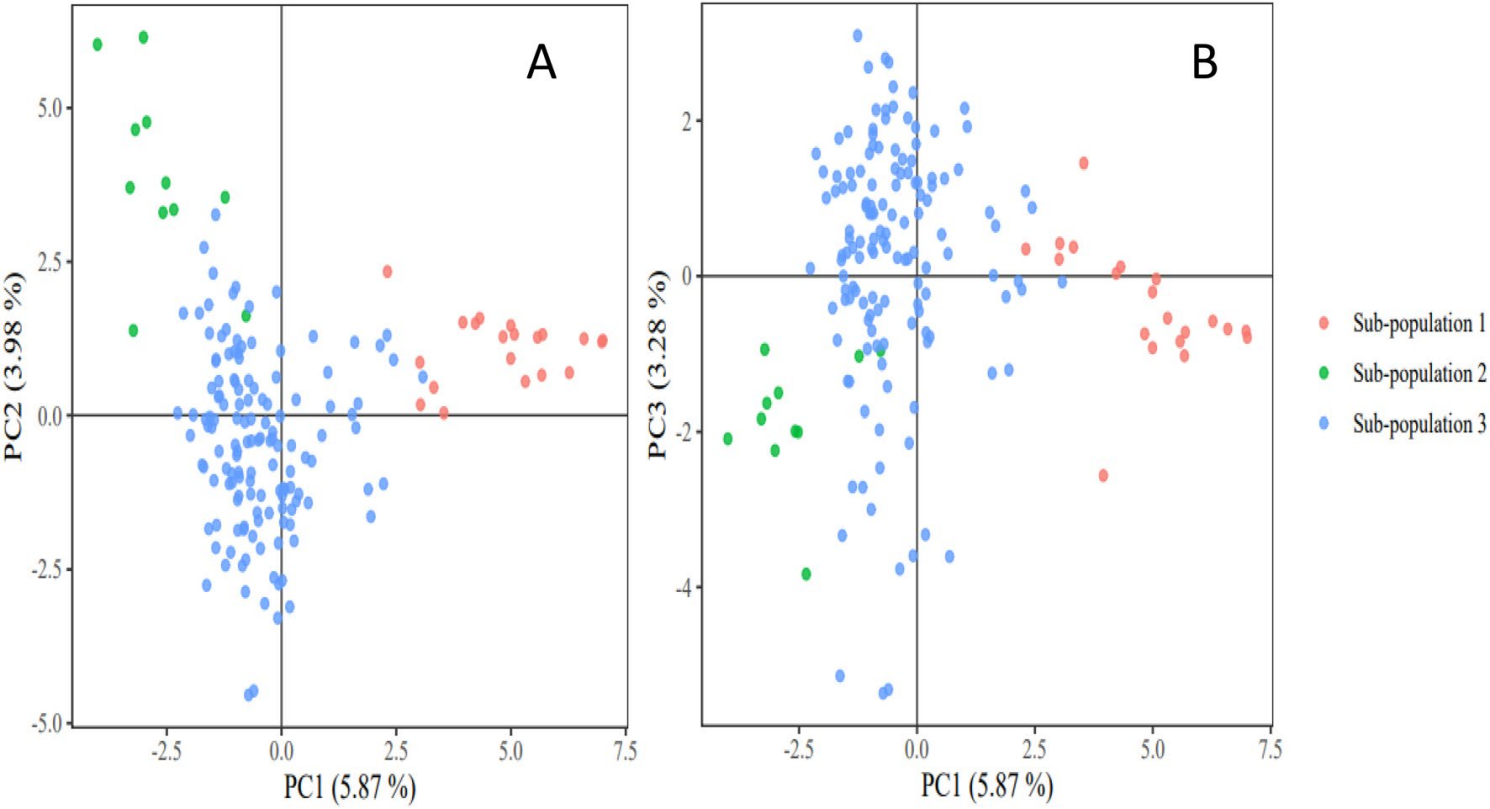
PC plots of 169 AYB accessions. The points are plotted along; **(A)** PC1 and PC2 **(B)** PC2 and PC3. The sub-populations are consistent with the population structure at K = 3. The PC plots were created using GenAlex version 6.501^61^.

The population structure of the accessions showed optimal delta K value = 2 and K = 3 (Supplementary Fig. S2). Based on the information from the hierarchical cluster, dendrogram delta K = 3 was selected as optimally describing the population structure within the accession. Thus, indicating three sub-populations within the 169 accessions (Fig. 2). The distribution of accessions into sub-populations followed the same pattern as the dendrogram clustering (Fig. 2). For example, the population structure showed 27 admixed individuals in sub-population 3; likewise, 3 accessions were admixed in sub-population 1, whereas 2 accessions were admixed in sub-population 2. Similarly, the principal coordinate analysis (PCoA) based on a pairwise genetic distance matrix across the 169 AYB accessions also split the accessions into three groups representing three sub-populations (Fig. 3). The PC1 axis represented 5.87% of the explained variation in the accessions, while the PC2 and PC3 axis explained 3.98% and 3.28% of the observed variation, respectively (Supplementary Table S4).

### Genetic diversity of identified sub-populations

Accessions in sub-population 3 were relatively genetically diverse, as shown by the number of unique alleles (154), Shannon information index (0.58 ± 0.004), expected heterozygosity (0.35 ± 0.003), observed heterozygosity (0.17), and % polymorphic loci (100%). In addition, sub-population 2 had the highest number (400) of unique alleles (private allele) in contrast to alleles in sub-population 1 (0) and sub-population 3 (154); similarly, sub-population 2 showed the highest number of effective alleles (1.64 ± 0.011) among the sub-populations. Sub-population 1 showed low values for all the estimated diversity parameters, being the least diverse; however, sub-population 3 was the most varied, followed by sub-population 2 (Table 3).

**Table 3:** Mean allelic patterns across three sub-populations.

Sub-population	1	2	3
Ne	1.39 ± 0.009	1.64 ± 0.011	1.60 ± 0.008
I	0.38 ± 0.006	0.56 ± 0.007	0.58 ± 0.004
He	0.23 ± 0.004	0.34 ± 0.004	0.35 ± 0.003
Ho	0.05	0.07	0.17
Number of private alleles (unique alleles)	0	400	154
% polymorphic loci	83.29%	89.44%	100%

*Ne* number of effective alleles, *I* Shannon’s information index, *He* expected heterozygosity, *Ho* observed heterozygosity.

Furthermore, the genetic distance among accessions in each sub-population revealed the existence of considerable genetic diversity in the studied materials. The distance matrix of accessions in Sub-population 1 ranged from 0.004 to 0.314 with a mean value of 0.194. The maximum distance in the sub-population was observed between accession TSs-431 and TSs-47, and the minimum distance was recorded between TSs-151B and TSs-449. In sub-population 2, the genetic distance between TSs-69 and TSs-95 was the highest (0.34), whereas TSs-109 and TSs-89 showed the least distance (0.14) in the sub-population. The average distance across the sub-population was 0.28. In addition, accessions in sub-population 3 presented a genetic distance that varied from 0.60 to 0.99 with an average of 0.71. TSs-60 and TSs-82 were the most diverse accessions based on their genetic distance. In contrast, a closer relationship was observed between TSs-166 and TSs-2015–07 than other accessions of the same population. Expected heterozygosity (He) was higher than the observed heterozygosity (Ho) in all the sub-populations viz; sub-population 1 (He = 0.23 ± 0.004, Ho = 0.05); sub-population 2 (He = 0.34 ± 0.004, Ho = 0.07) and sub-population 3 (He = 0.35 ± 0.003, Ho = 0.17) an indication of inbreeding.

### Analysis of molecular variance (AMOVA)

The calculated distance was used to analyze molecular variance (AMOVA). The AMOVA performed on the three sub-population identified by STRUCTURE revealed that 13% of the total variation was found among populations, whereas the remaining 87% was found among individuals (Supplementary Fig. S3). The pairwise *F*_*ST*_ among the three sub-populations varied from 0.14 to 0.39 and were significant at P-value (0.001), while the *F’*_*ST*_ ranged from 0.12 to 0.28. A high level of differentiation was observed among accessions in sub-population 1 and sub-population 2 (0.39). Additionally, the level of differentiation observed between sub-populations 1 and 3 (0.20) was slightly higher than that observed between sub-populations 2 and 3 (0.18). (Table 4).

**Table 4:** Pairwise *F*_*ST*_, *P,* and *F’*_*ST*_ of the three sub-populations of 169 AYB accessions of IITA’s collection.

Sub-populations	*F*_*ST*_	*P*	*F’*_*ST*_
Between sub-populations 1 and 2	0.39	0.001	0.28
Between sub-populations 1 and 3	0.20	0.001	0.13
Between sub-populations 2 and 3	0.18	0.001	0.12
Between the three sub-populations	0.14	0.001	0.20

*F*_*ST*_  fixation index = (variance among population/total variance), *P* significance level, *F’*_*ST*_ standardized fixation index = *(F*_*ST*_/*F*_*ST*_ max).

### Combined analysis of phenotypic and genotypic data

**Figure 4:**
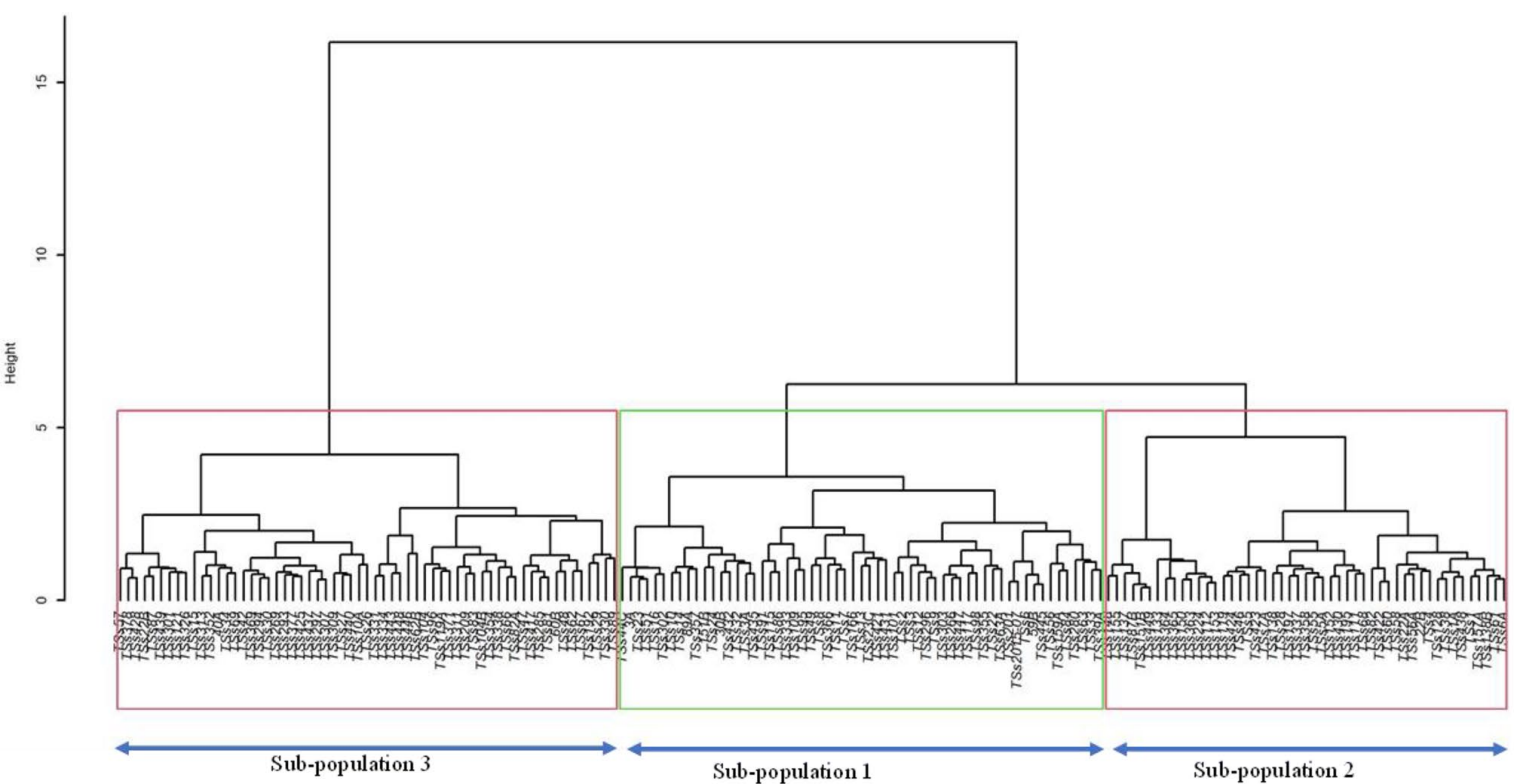
Hierarchical cluster dendrogram generated from the combined distant matrixes of phenotypic and genotypic data. The dendrogram was created in R software version 4.1.156 based on Ward.D2.

**Figure 5:**
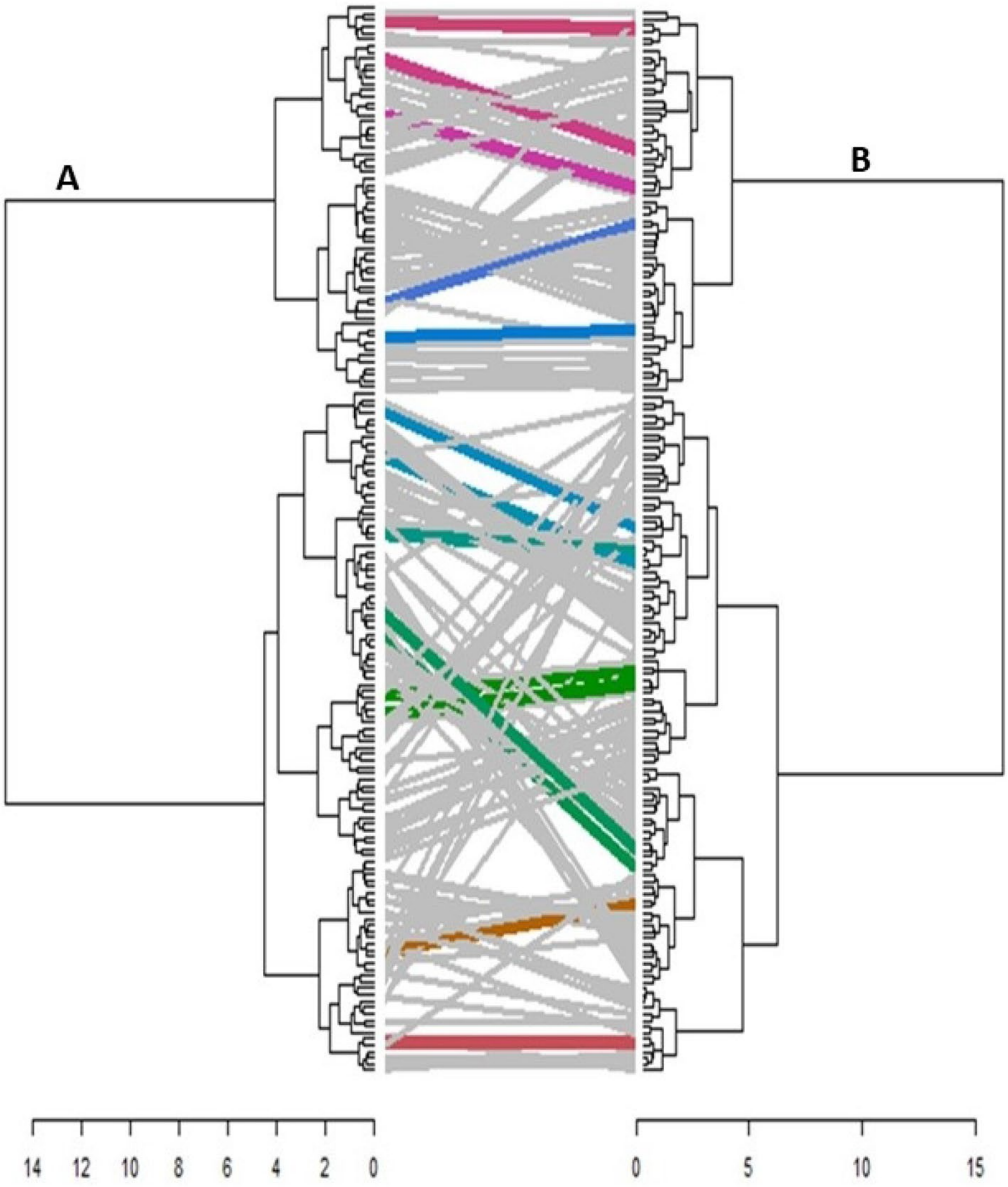
A comparison between hierarchical dendrograms generated from **(A)** phenotypic data and **(B)** combined data. The colored lines running through the two dendrograms represent accessions that clustered together both in the phenotypic and combined dendrogram. In contrast, the non-colored lines represent accessions that did not retain their position in both dendrograms. The dendrogram comparison was achieved using R software version 4.1.1^56^.

The distance matrix of the combined phenotypic and genotypic data revealed a maximum genetic distance of 0.89, observed between TSs-446 and TSs-363. The minimum distance, 0.12, was displayed between TSs-151B and TSs-87B, while the average distance across the accessions was 0.56. A hierarchical cluster generated from the summation of the phenotypic and genotypic distances revealed three clusters representing three sub-populations (Fig. 4). Sub-population 3 had the highest number of accessions (61), which was followed by sub-population 1 (59) and sub-population 2 (49). The high cophenetic coefficient of correlation (0.84) reported for the combined matrix further confirms the goodness of fit of the combined hierarchical cluster dendrogram. The grouping of accessions based on phenotypic, genotypic, and combined (phenotypic and genotypic) analysis showed that most accessions remained together in a cluster across the different dendrograms. Comparing the dendrogram drawn with the phenotypic data to the dendrogram drawn with the combined (phenotypic and genotypic) data (Fig. 5), sub-population (cluster) 3 of the combined dendrogram corresponds to sub-population (cluster) 2 of the phenotypic dendrogram. All 61 accessions were present in both clusters of the different dendrogram. Similarly, 49 of the 59 accessions in sub-population (cluster) 1 of the phenotypic dendrogram were also found in sub-population 1 of the combined dendrogram. Also, 26 of the 36 accessions grouped in sub-population (cluster) 3 were likewise grouped in sub-population 2 of the combined dendrogram. Additionally, out of the 61 accessions originally grouped in sub-population 3 of the combined dendrogram, 56 remained together in sub-population 3 of the genotypic dendrogram, while 4 accessions (TSs-417, TSs-69, TSs-83, and TSs-89) grouped in sub-population 2 of the genotypic dendrogram. Again, of the 59 accessions originally grouped in sub-population 1 of the combined dendrogram, 51 maintained their membership together in sub-population 3 of the genotypic dendrogram while 6 accessions (TSs-109, TSs-115, TSs-333, TSs-86, TSs-91, and TSs-95) all grouped in sub-population 2 of the genotypic dendrogram. The comparison between the phenotypic dendrogram and the genotypic dendrogram showed that some of the accessions maintained the same clustering pattern in both dendrograms. Many accessions (62 out of 72) grouped in sub-population 1 of the phenotypic dendrogram remained together and were found in sub-population 3 of the genotypic dendrogram. The rest 10 accessions grouped in sub-population 1 (TSs-138, TSs-355, TSs28 and TSs-358) and sub-population 2 (TSs-333, TSs-95, TSs-4, TSs-109, TSs-115 and TSs-86).

The Mantel test revealed a low correlation (r = 0.02); between the dissimilarity matrixes of phenotypic and genotypic data; however, the correlation r = 0.22 observed between genotypic and combined matrixes suggests that the matrix entries are moderately associated. A high positive association r = 0.96 was observed between the combined matrix and phenotypic matrix (Supplementary Table S5; Supplementary Fig. S5). The mean analysis of the three sub-population generated from the combined (phenotypic + genotypic) shows that accessions in sub-population 1 reached 50% flowering (117.79 days) earlier than accessions in sub-population 2 (121.70 days) and sub-population 3 (118.26 days) and was significantly different from sub-population 2. Also, accessions grouped in sub-population 3 germinated earlier (12.32 days) and significantly differed from those in sub-population 2 (12.84 days). Furthermore, accessions in sub-population 1 yielded more seeds (67.31 g) than accessions in sub-population 2 (56.28 g) and 3 (61.14 g), and the mean value was significantly different from that of sub-population 2. Across the three sub-populations, accessions grouped in sub-population 2 showed more diversity in flower color (2.02) and were significantly different from accessions in sub-population 3 (1.92). The diversity in seed color was also more prominent in sub-population 2 than observed in sub-populations 1 and 3. Moreso, a reasonable number (49%) of accessions clustered in sub-population 3 showed no variegation on seed, and the sub-population was significantly different from sub-populations 1 and 2. Similarly, 33% of accessions in sub-population 3 exhibited pod-shattering; the sub-population was significantly different from sub-population 1 and 2. Although the diversity parameters of the genotypic data varied across sub-population, an estimate of heterozygosity showed that sub-population 2 were more diverse than other sub-populations; again, the SNP markers associated with accessions in sub-population 2 showed 100% polymorphic loci, which was followed closely with markers associated with accessions in sub-population 3 (99.94%) and those in sub-population 1 (99.27%) (Table 5).

**Table 5:** Means and standard deviation of 26 phenotypic traits and genotypic diversity indices of three sub-populations identified based on the combined (phenotypic + genotypic) distant matrix.

Phenotypic traits	Sub-population 1		Sub-population 2		Sub-population 3				
	Mean	SD	Mean	SD	Mean	SD			
**Quantitative**									
Days to 1st flowering	96.09a	4.05	96.84a	4.19	95.99a	4.76			
D50FL	117.79b	6.18	121.70a	6.53	118.26b	4.98			
Days to germination	12.39b	0.78	12.84a	0.78	12.32b	0.64			
Dry seed matter (%)	92.98a	0.88	93.33a	0.84	93.23a	0.81			
NSDPD	12.19a	1.09	11.67b	1.00	12.08ab	1.03			
Pod length (cm)	16.94a	1.12	15.84b	1.09	16.86a	1.21			
Petiole length (cm)	4.63ab	0.41	4.46b	0.40	4.64a	0.39			
Seed length (mm)	7.95a	0.77	7.61b	0.29	7.81ab	0.39			
SDMC (%)	7.02a	0.88	6.67a	0.84	6.77a	0.82			
Seed thickness (mm)	6.12a	0.23	6.11a	0.22	6.10a	6.10			
Seed width (mm)	6.04a	0.68	6.12a	0.71	6.07a	0.49			
Total germination	7.26a	1.04	6.95a	1.13	7.04a	1.03			
TLL (cm)	9.89a	0.83	9.45ab	0.73	9.94a	0.91			
TLW (cm)	3.65a	0.34	3.48b	0.37	3.77a	0.35			
Total seed weight (g)	67.31a	26.42	56.28b	16.60	61.14ab	22.91			
100 seed weight (g)	19.80a	2.41	19.07a	1.49	19.39a	1.64			
**Qualitative (ordinal)**									
Flower colour	1.93ab	0.25	2.02a	0.322	1.92b	0.331			
Seed colour	1.93a	1.61	2.02b	0.602	1.92c	1.76			
Seed shape	1.93a	0.64	2.02a	0.71	1.92a	0.62			

Qualitative (binary)	No. present	No. absent	Sig	No. present	No. absent	Sig	No. present	No. absent	Sig
MASPIG	0	59	a	1	48	a	61	0	b
BRAPIG	0	59	a	0	49	a	60	1	b
PETPIG	0	59	a	0	49	a	61	0	b
GHABIT	37	22	a	2	47	b	34	27	c
Seed variegation	12	47	a	25	24	b	12	49	c
Pod morphology	18	41	a	1	48	b	15	46	a
Pod shattering	11	35	a	0	49	a	33	28	b

Genotypic traits	Mean	SE	Mean	SE	Mean	SE			
Observed heterozygosity	0.15	0.002	0.16	0.002	0.13	0.002			
Expected heterozygosity	0.33	0.003	0.35	0.003	0.36	0.003			
% polymorphic loci	99.27		100.00		99.94				

*SD* standard deviation, *D50FL* days to 50% flowering, *NSDPD* number of seeds per pod, *SDMC* seed moisture content, *TLL* terminal leaf length, *TLW* terminal leaf width, *MASPIG* main stem pigmentation, *BRAPIG* branch pigmentation, *PETPIG* petiole pigmentation, *GHABIT* growth habit, *SE* standard error, *No* number, *sig* significance.

## Discussion

Despite the food and nutrition potentials of AYB, farmers’ interest in cultivating the crop is perceived to be dwindling^10,12^; the lack of interest could be linked to identified limitations, including prolonged cooking time of about 6–24 h, the abundance of anti-nutritional factors in seeds, and an extended maturity cycle of about 9–10 months. Understanding the population structure and identifying genetic variations within the crop’s germplasm can facilitate its improvement^18^. Phenotypic and molecular methods are widely explored for genetic study in plant species^18,37^, neither of the methods is superior to the other^29^. The methods can, therefore, be used independently or complementary^31^. The present study used DArTseq derived SNPs and combined approach to study the genetic diversity and population structure of a selected AYB germplasm.

The significance of PCA in studying the extent and pattern of variations across populations has been documented by authors Sharma et al.^38^; Nadeem et al.^15^. Previous characterization studies in AYB likewise reported the relevance of phenotypic traits in understanding genetic diversity in the crop^17,32^. In the present study, analysis based on phenotypic traits indicated a substantial diversity within the accessions. PC1 to PC8 accounted for 68.68% of the phenotypic variability observed in the accessions. In particular traits, including days to 1st flowering, days to 50% flowering, dry seed matter, petiole length, 100 seed weight, and seed color contributed highly to the observed variations as shown by their PC values and contribution to more than one PC axis. The mentioned traits can be used to assess diversity in AYB collections efficiently. A genetic distance range of 0.06–0.57 was observed in the present study and the accessions clustered into three sub-populations. In similar studies using phenotypic traits, Aina et al.^17^ obtained a distance of 0.0003–0.59 across 50 AYB collections sourced from IITA. The variation across means of phenotypic traits, e.g., days to 1st flowering (95.31–98.67 days), days to 50% flowering (117.17–124.33 days), total seed weight (53.06–66.39 g), observed in our study is an indication of the existing diversity in the crop. However, the mean values reported for days to 1st flowering and days to 50% flowering differs from earlier findings; in the phenotypic evaluation of 16 AYB accessions grown in Nigeria, days to 1st flowering was reported to vary from 139.40 to 159.21 days^35^. Also, Aina et al.^17^ obtained mean values between 65.00 and 97.00 for days to 50% flowering in 50 accessions characterized in Nigeria. Similarly, Ojuederie et al.^32^ reported days to 50% flowering as between 97.50 and 115.83 across 40 accessions evaluated in Nigeria. Nevertheless, the differences between our findings and previous studies could be due to variations in environmental conditions and sample size.

In addition, the correlation among 26 traits phenotypic traits in the present study showed significant associations across most of the traits; for instance, days to 1st flowering showed a significant positive correlation with days to 50% flowering (0.67), which is a good indication towards breeding for early maturity. The availability of accessions with less than 9–10 months maturity could encourage the crop's cultivation by farmers. Seed moisture content correlated positively with total seed weight (0.58), showing the importance of trait in assessing seed yield. Positive correlations between seed weight and other characteristics were also reported in earlier studies^14,34,35^. Accessions including TSs-2015-07, TSs-1, TSs-12, TSs-10, and TSs-109 found in sub-population 1 characterized with reduced days to 50% flowering (117.17) could be choice materials for breeding of early maturity in the crop. Sub-population 1 was likewise associated with high seed yield (66.93 g) and number of seeds per pod (12.18) and could therefore be exploited for improving seed yield in the crop. The selection of such materials for improvement has been recommended as an important improvement strategy for the crop^39,40^. Also, non-shattering accessions in sub-population 1 could be useful in breeding for accessions with reduced pod shattering. Same with our findings, TSs-1 and TSs-12 were also identified as non-pod shattering accessions^39^. Furthermore, improved cultivars could be developed from hybridizing the distantly related accessions (TSs-363 and TSs-446) identified in this study by phenotypic and genotypic analysis (TSs-431 and TSs-47). Past genetic diversity studies in AYB using AFLP, RAPD, ISSR, and SSR markers transferred from cowpea reported considerable diversity in the crop^33–35^. Among the three sub-population observed, sub-population 3 was the most genetically diverse of the three sub-populations followed closely by sub-population 2 and then sub-population 1 as indicated by the population’s high expected heterozygosity (He), Shannon information index (I), and percentage polymorphic loci (PIC). Across the three subpopulations, the observed heterozygosity was lower than the expected heterozygosity, which can be attributed to the non-random mating among the individuals of the population suggesting inbreeding. The finding could be explained by the fact that AYB shows a high percentage of self-pollination^2,3^. The SNPs dependent approaches, STRUCTURE, hierarchical cluster dendrogram, PCoA, and AMOVA implemented in the present study consistently identified three subpopulations across the 169 AYB accessions. The consistency in the clustering pattern agrees with reports in Camelina^41^ rice^42^, and cowpea^43^. The genetic differentiation among the three sub-populations was significant was significant (P < 0.001) and the fixation index ranged from (*F*_*ST*,_ 0.14–0.39), indicating a medium to a high amount of genetic differentiation^42,44^. Therefore accessions from each sub-population can be crossed and tested for heterosis.

In the present study, the combined genetic distance generated from phenotypic and genotypic data also indicated three sub-populations. The high cophenetic correlation coefficient ≥ 7.0 observed across the three distance matrixes used in constructing each hierarchical cluster dendrogram shows each dendrogram’s fitness and ruling distortion in the data. Subjectively, the degree of fit is interpreted as: 0.9 ≤ r, very good fit; r < 0.7, very poor fit^45,46^. The Mantel test, Mantel^47^, showed a low correlation between the phenotypic and genotypic distance matrix, similar to findings reported in the diversity analysis of pepper^44^ and winged yam^18^. The absence of a strong association between the phenotypic and genotypic data could be because the SNP data are not associated with the phenotypic traits evaluated; it could also be because molecular markers generally detect the non-adaptive types of variation and are not subjected to either/both natural and artificial selection which is attributed to phenotypic traits^18,48^. However, due to the inconsistency observed in studies involving phenotypic and genotypic evaluations, authors have recommended combining genotypic and phenotypic data as the best option for the efficiency of diversity assessment^48–50^. The evaluation of the grouping of accessions in the three dendrograms (phenotypic, genotypic, and combined) revealed a high pattern of similarity. The accessions grouped in sub-population 3 of the combined dendrogram retained 100% of their membership in sub-population 2 of the phenotypic dendrogram. Also, 83% of the accessions in sub-population 1 of the combined dendrogram clustered together in sub-population 1 of phenotypic dendrogram; however, the remaining 7% grouped in sub-population 3 of the phenotypic dendrogram. Similarly, 86% of the accessions in sub-population 1 of the phenotypic dendrogram remained together in sub-population 3 of the genotypic dendrogram, while 14% of the accessions clustered in sub-population 2 and 3 of the genotypic dendrogram. Furthermore, 86% of the accessions grouped in sub-population 1 of combined dendrogram maintained their membership in sub-population 3 of the genotypic dendrogram. The high correlation between phenotypic and combined dendrogram observed in this research is similar to the findings in winged yam^18^. However, the level of correlation obtained between the genotypic and combined dendrogram differs from the reported in winged yam^18^.

In our study, the genetic diversity across the AYB population was confirmed further by the presence of high polymorphic loci of SNP markers associated with each population across the combined analysis. For example, sub-population 2 showed 100% polymorphic loci; more so, high heterozygosity was visible in sub-population 2, indicating high genetic diversity.

Conclusively, a sufficient level of genetic diversity was revealed among and within the 169 AYB accessions evaluated with phenotypic descriptors, DArT-SNPs markers, and combined analysis. The correlations observed between traits, including early maturity, seed yield, and main stem pigmentation, are valuable for AYB breeding activities. The polymorphic DArT-SNPs markers likewise showed efficiency in detecting the population structure and genetic diversity; the markers can therefore be explored for use in genome-wide association study (GWAS) and marker-assisted selection (MAS) in AYB. The complementary approach of combining phenotypic and genotypic data can be implemented in selecting divergent parental materials for hybridization, marker-assisted selection (MAS), and genome-wide association study (GWAS).

## Materials and method

### Plant material

A total of 169 AYB accessions sourced from the GenBank of the International Institute of Tropical Agriculture (IITA) were evaluated for the present study; the passport data of the materials are shown in Supplementary Table S1. The AYB accessions were sourced and received following all the rules guiding plant material transfer between Nigeria and Ethiopia.

### Phenotypic characterization

The 169 accessions were planted over two cropping seasons (2019/2020; 2020/2021) at Jimma Agricultural Research Center (JARC), Jimma, Ethiopia. The field evaluation was carried out under regulations guiding field experimentation of JARC. The experimental field sits at 1739 masl, N07°39.962′, and E036°46.74′ and was laid in Alpha lattice design with two replications of ten plants per accession. After sowing, each plant was stalked with a 3 m stick. Each accession was characterized using 26 phenotypic traits (16 quantitative and 10 qualitative); the traits were selected based on their abilities to comprehensively capture the existing diversity through all the crop’s vital developmental stages to yield attributes. The IITA AYB descriptor list guided the trait selection^51^. The phenotypic traits evaluated, the assessment period and the method are presented in Supplementary Table S2.

### DArT sequencing

Two weeks after planting, about 1 g of young, healthy leaves was collected into labeled 1.2 ml cluster tubes. The tubes were immediately capped, placed on an ice bucket, and transferred to the Plant Molecular Laboratory at Jimma University, where they were kept in −80 °C freezer before lyophilization. The lyophilized leaves were shipped to SEQART Africa Laboratory at International Institute of Tropical Agriculture (ILRI), Nairobi, for DNA extraction and genotyping. The genomic DNA was extracted using the NucleoMag Plant kit, and DNA was purified with genomic DNA clean and concentrator. The purified DNA was quantified on 0.8% agarose gel electrophoresis. The DArT genotyping was done using SEQART Africa genotyping protocol^52^. In brief, genomic DNA was digested with two restriction enzymes; Mst1 was used as the rare cutter and pst1 as the frequent cutter. The digested DNA fragments were ligated using a common adapter, and a barcode adapter, the DNA fragments with a combination of common and barcoded adapters were selectively amplified. The PCR products were pooled and purified using a QIAquick PCR purification kit. The purified PCR products were sequenced on Illumina Hiseq 2500 using single reads. After the sequencing, FASTQ files generated by DArT were aligned against the African yam bean draft genome unpublished (provided by the Biosciences Eastern and Central Africa (BeCA-ILRI), and a HapMap file was generated.

### Multivariate analysis and cluster generation of phenotypic data

The phenotypic data were analyzed with the R statistical package (Version 4.1.1)^53^. Analysis of variance (ANOVA) for each quantitative trait across two years was calculated using the PBIB.test function from the Agricolae R statistical package. Tukey’s HSD test was performed to test the significant difference among the means. The ANOVA was performed using

$${\text{the model}}:{\text{ Y}}_{{{\text{ijkl}}}} = \, \mu \, + {\text{ B }}\left( {\text{E}} \right)_{{{\text{j}}({\text{i}})}} + {\text{ G}}_{{\text{k}}} + {\text{ GE}}_{{{\text{ij}}}} + {\text{ e}}_{{{\text{ijkl}}}}$$

where Y is the traits, µ is the grand mean, E is the environment effect (years), B(E) is the block effect in environment, G is the genotype effect, GE is the genotype by environment interaction, e is the error. Furthermore, means analysis for qualitative data (ordinal) was analyzed using the Kruskal–Wallis test, and a post-hoc Dunns test was performed to test the significance of the means. The (binary data) were analyzed using the Chi-square test. Principal component analysis (PCA) across the LSmeans of phenotypic traits generated from the genotype by environment analysis was computed using the PCAmix function from the PCAmixdata package. PCAmixdata is a suitable R package for multivariate qualitative and quantitative data analysis. The daisy function from the cluster package was used to generate the dissimilarity matrix using Gower^54^ distance method, while the phylogenetic and evolution (ape) package was used to construct the hierarchical cluster dendrogram using the Ward.D2 option. The goodness-of-fit of the hierarchical dendrogram was estimated using the cophenetic coefficient of correlation. Finally, the correlation among the phenotypic traits (qualitative) was performed using the GoodmanKruskal package. The ChartCorrelation function from the PerformanceAnalytics package was used for the quantitative traits.

### Analysis of molecular data

A total of 7930 SNPs were generated from the DArTseq. The HapMap file was loaded into TASSEL software 5.2.73^55^ for further filtering. The filtering was performed on sites retaining SNPs with a maximum of 20% missing values and a minimum and maximum allele frequency of 0.05 and 0.95, respectively. The filtered data generated 1789 SNPs, and the major allele and minor allele frequency were generated for the 1789 SNPs. The pairwise dissimilarity matrix, Identity-by-state (IBS) matrix, was calculated among individuals using PLINK software^56^. The IBS matrix was inputed into R software version 4.1.1^53^, and the ape package was used to construct a hierarchical cluster dendrogram based on Ward.D2 option. The effect of outliers in the pairwise matrix was minimized by using the cophenetic coefficient of correlation analysis implemented in R to estimate the goodness-of-fit of the hierarchical cluster dendrogram.

The population structure analysis of the 169 AYB accessions was performed using STRUCTURE software version 2.34 (Jul 2012)^57^. First, the parameter set was inputed as follows; length of run, 30,000, and number of Markov chain Carlo (MCMC) after burning 30,000. Secondly, the “Admixture model” option of the “Ancestry Model” was selected; the admixture model is known to detect historical population admixture and estimate the number of natural genetic clusters. Next, the possible sub-population was estimated with a K-value analysis of k1 to k10; for each simulation, k was independently repeated five times. Finally, the STRUCTURE HARVESTER^58^ was implemented, and Evanno’s Delta K^59^ option was used to estimate the appropriate K value to describe the likely sub-population in the data set.

GenAlex software version 6.501^60,61^ was used in calculating basic diversity parameters, including the number of private alleles, the number of effective alleles (Ne), Shannon information index (I), observed heterozygosity (Ho), expected heterozygosity (He), and fixation index (F) and % polymorphic loci across the 169 accessions and each sub-population. The clustering pattern of accessions was validated using principal component analysis (PCoA) implemented in GenAlex. The pairwise population differentiation statistics (F_ST_), standardized (F’_ST_), and Shannon index of the observed populations were generated using analysis of molecular variance (AMOVA) implemented in GenAlex.

### Combined phenotypic and genotypic analysis

The IBS distance matrix generated from the genotypic evaluation and the Gower distant matrix generated from the phenotypic evaluation were loaded into R. The R package Dendextend was used to generate a combined genetic distance by summing the phenotype distance matrix and genotype distance matrix. The combined distance matrix was used to construct a hierarchical cluster dendrogram based on the Ward.D2 method. The cophenetic coefficient of correlation was used to measure the accuracy of the hierarchical cluster dendrogram.

Furthermore, the dendrograms generated from the phenotypic, genotypic and combined evaluation were compared against each other using the R package Dendextend. The significance between the phenotypic matrix and the genotypic matrix, phenotypic matrix versus the combined matrix, and genotypic matrix versus the combined matrix was estimated using the Monte-Carlo option of the Mantel test^47^ with 9999 permutations. Similarly, the clusters generated from the combined dendrogram were inputed as variables for ANOVA. Finally, the significance of the cluster means was ascertained through Tukey’s HSD Post-Hoc test.

## Supplementary Information

Supplementary Information.

## Data Availability

The data set generated during an/or analyzed during the current study are available from the corresponding author on resonable request.
