# Supplementary material for: Genetic diversity and population structure of an African yam bean (Sphenostylisstenocarpa) collection from IITA GenBank
Source: Sci Rep. 2022 Mar 15;12:4437. doi: 10.1038/s41598-022-08271-4 (PMC8924269; doi:10.1038/s41598-022-08271-4)
Supplement: Supplementary file 1 — Supplementary Information. [file 41598_2022_8271_MOESM1_ESM.pdf]

Manuscript Title: Genetic Diversity and Population Structure of An African yam bean (*Sphenostylis stenocarpa*) Collection from IITA GenBank

Authors: Ndenum Suzzy Shitta*, Nnanna Unachukwu, Alex Chukwudi Edemodu, Abush Tesfaye Abebe, Happiness O Oselebe, Wosene Gebreselassie Abtew *

Supplementary Table S1. Passport data of 169 AYB accessions used in the present study

S/N	Accession	Country of Origin	S/N	Accession	Country of Origin
1	TSs-1	Nigeria	53	TSs-82	Nigeria
2	TSs-10	Nigeria	54	TSs-84	Nigeria
3	TSs-109	Nigeria	55	TSs-86	Nigeria
4	TSs-11	Nigeria	56	TSs-9	Nigeria
5	TSs-111	Nigeria	57	TSs-91	Nigeria
6	TSs-112	Nigeria	58	TSs-95	Nigeria
7	TSs-115	Nigeria	59	TSs-98	Nigeria
8	TSs-12	Nigeria	60	TSs-15	Nigeria
9	TSs-13	Nigeria	61	TSs-26	Nigeria
10	TSs-137	Nigeria	62	TSs-27	Nigeria
11	TSs-138	Nigeria	63	TSs-29	Nigeria
12	TSs-14	Nigeria	64	TSs-30	Nigeria
13	TSs-148	Nigeria	65	TSs-34	Nigeria
14	TSs-150	Nigeria	66	TSs-42	Nigeria
15	TSs-152	Nigeria	67	TSs-47	Nigeria
16	TSs-153	Nigeria	68	TSs-48	Nigeria
17	TSs-155	Nigeria	69	TSs-57	Nigeria
18	TSs-16	Nigeria	70	TSs-62	Nigeria
19	TSs-2	Nigeria	71	TSs-81	Nigeria
20	TSs-22	Nigeria	72	TSs-83	Nigeria
21	TSs-23	Nigeria	73	TSs-87	Nigeria
22	TSs-24	Nigeria	74	TSs-89	Nigeria
23	TSs-28	Nigeria	75	TSs-96	Nigeria
24	TSs-3	Nigeria	76	TSs-66	Bangladesh
25	TSs-32	Nigeria	77	TSs-67	Bangladesh
26	TSs-33	Nigeria	78	TSs-68	Ghana
27	TSs-38	Nigeria	79	151B	Not Available
28	TSs-4	Nigeria	80	22B	Not Available
29	TSs-46	Nigeria	81	30B	Not Available
30	TSs-49	Nigeria	82	3A	Not Available
31	TSs-5	Nigeria	83	59B	Not Available
32	TSs-51	Nigeria	84	89A	Not Available
33	TSs-55	Nigeria	85	TSs-101	Not Available
34	TSs-56	Nigeria	86	TSs-117	Not Available
35	TSs-58	Nigeria	87	TSs-119	Not Available
36	TSs-6	Nigeria	88	TSs-128	Not Available
37	TSs-60	Nigeria	89	TSs-133	Not Available
38	TSs-61	Nigeria	90	TSs-138B	Not Available
39	TSs-63	Nigeria	91	TSs-151B	Not Available
40	TSs-7	Nigeria	92	TSs-157A	Not Available
41	TSs-8	Nigeria	93	TSs-159A	Not Available
42	TSs-166	Not Available	94	TSs-358	Not Available
43	TSs-195	Not Available	95	TSs-56A	Not Available
44	TSs-197	Not Available	96	40A	Not Available
45	TSs-1A	Not Available	97	60B	Not Available
46	TSs-201	Not Available	98	TSs-104	Not Available
47	TSs-2015-07	Not Available	99	TSs-104B	Not Available
48	TSs-224	Not Available	100	TSs-10A	Not Available
49	TSs-22A	Not Available	101	TSs-119A	Not Available
50	TSs-23C	Not Available	102	TSs-121	Not Available
51	TSs-266	Not Available	103	TSs-192	Not Available
52	TSs-280	Not Available	104	TSs-209	Not Available

S/N	Accession	Country of Origin	S/N	Accession	Country of Origin
105	TSs-296	Not Available	138	TSs-22B	Not Available
106	TSs-302	Not Available	139	TSs-269	Not Available
107	TSs-303	Not Available	140	TSs-285	Not Available
108	TSs-333	Not Available	141	TSs-293	Not Available
109	TSs-337	Not Available	142	TSs-294	Not Available
110	TSs-355	Not Available	143	TSs-297	Not Available
111	TSs-357	Not Available	144	TSs-301	Not Available
112	TSs-363	Not Available	145	TSs-309	Not Available
113	TSs-364	Not Available	146	TSs-311	Not Available
114	TSs-365	Not Available	147	TSs-313	Not Available
115	TSs-366	Not Available	148	TSs-326	Not Available
116	TSs-39A	Not Available	149	TSs-330	Not Available
117	TSs-3A	Not Available	150	TSs-331	Not Available
118	TSs-421	Not Available	151	TSs-338	Not Available
119	TSs-422	Not Available	152	TSs-352	Not Available
120	TSs-423	Not Available	153	TSs-354	Not Available
121	TSs-424	Not Available	154	TSs-369	Not Available
122	TSs-430	Not Available	155	TSs-371	Not Available
123	TSs-431	Not Available	156	TSs-377	Not Available
124	TSs-432	Not Available	157	TSs-378	Not Available
125	TSs-435	Not Available	158	TSs-417	Not Available
126	TSs-438	Not Available	159	TSs-425	Not Available
127	TSs-445	Not Available	160	TSs-428	Not Available
128	TSs-447	Not Available	161	TSs-437	Not Available
129	TSs-449	Not Available	162	TSs-439	Not Available
130	TSs-44C	Not Available	163	TSs-443	Not Available
131	TSs-5A	Not Available	164	TSs-446	Not Available
132	TSs-63A	Not Available	165	TSs-448	Not Available
133	TSs-6A	Not Available	166	TSs-62B	Not Available
134	TSs-7A	Not Available	167	TSs-69	Not Available
135	TSs-87B	Not Available	168	TSs-82A	Not Available
136	TSs-307	Not Available	169	TSs-334	Not Available
137	TSs-440	Not Available			

Supplementary Table S2. Quantitative and qualitative traits characterized, assessment period, and method used during the study

S/N	Quantitative traits	Assessment period	Method
1	Days to 50% flowering	Date of planting to date when 50% of the plant reached flowering	Count of number of days from date of first flowering till date 50% of plants blossomed
2	Days to 1st flowering	Date of planting to date when a plant produces flower	Count of number of days from planting date to date taken for a plant to blossom
3	Days to germination	11 days after sowing	Count of number plants that germinated for each accession
4	Seed moisture content (%)	Postharvest time	Measured using grain moisture meter (Draminski GMM)
5	Number of seeds per pod	Postharvest	Count of number of seeds in each pod
6	Petiole length (cm)	100 days after planting	Measured from the base to the point where the three leaflets join using a ruler
7	Pod length (cm)	Postharvest time	Measured from the peduncle stalk end to pod beak end using a ruler
8	Seed length (mm)	Postharvest time	Measured with the aid of a Vernier caliper (APCO)
9	Seed thickness (mm)	Postharvest time	Measured with the aid of a digital Vernier caliper (APCO)
10	Seed width (mm)	Postharvest time	Measured with the aid of a digital Vernier caliper (APCO)
11	Terminal leaf length (cm)	100 days after planting	Measured from the pulvinus to the apical end sing a ruler
12	Terminal leaf width (cm)	100 days after planting	Measured along the widest part of the terminal leaflet using a ruler
13	Dry seed matter (%)	Postharvest time	100-seed moisture content value
14	Total seed weight (g)	Postharvest time	Mass of seeds from each accession was measured with an electronic weighing scale (Develo)
15	Total germination	11 - 27 days after sowing	Count of number of all plants that sprang up for each accession
16	100 seed weight (g)	Postharvest time	Mass of randomly selected 100 seeds were measured using an electronic weighing scale (Develo)
	Qualitative traits		
1	Growth habit	After 50% flowering	Visual scoring of plants on a scale of 1-2; 1= erect type; 2 = bushy type
2	Pod morphology	Postharvest time	Visual assessment of pods for presence or absence of seed cavity on pods. On a scale of 1-2; 1= presence, 0 = absence
3	Pod shattering	At harvest time	Visual inspection of pods that release their seeds upon maturity
4	Flower color	7 days after 50% flowering	Visual examination using methue color guide. On a scale of 1-3; 1 = (11A4) pink rose/ pale red; 2 = 12A2 (reddish or pinkish white); 3 = 12C3 (greyish ruby/ purple)
5	Seed shape	Postharvest time	Visual scored on a scale of 1- 3. 1= round; 2 = oval; 3 = oblong.
6	Seed color	Postharvest time	Visual scoring using a color guide. Scale 1-5 was used. 1 = brown, 2 = grey, 3 = black, 4 = brown black, 5 = grey black, 6 = black grey

7	Main stem pigmentation	7 days after 50% flowering	Visual scoring of the presence or absence of coloration. 1= presence, 0 = absence
8	Branch pigmentation	7 days after 50% flowering	Visual scoring of the presence or absence of coloration. 1= presence, 0 = absence
9	Petiole pigmentation	7 days after 50% flowering	Visual scoring of the presence or absence of coloration. 1= presence, 0 = absence
10	Seed variegation	Postharvest time	Visual scoring of spots/dots on seeds on a scale of 0-1. 1= presence; 0 = absence

Supplementary Table S3. Phenotypic traits variations and diversity indices for sub-populations

Phenotypic	Sub-population 1		Sub-population 2		Sub-population 3	
Quantitative traits	Average	SD	Average	SD	Average	SD
Days to 1st flowering	95.31b	3.73	95.99b	4.76	98.67a	3.97
Days to 50% flowering	117.17b	5.67	118.26b	4.98	124.33a	5.74
Days to germination	12.44b	0.75	12.32b	0.64	12.89a	0.84
Dry seed matter (%)	92.10b	0.85	93.23ab	0.82	93.43a	0.87
Number of seed per pod	12.18a	1.14	12.08a	1.03	11.50b	0.78
Pod length (cm)	16.77a	1.02	16.86a	1.20	15.77b	1.36
Petiole length (cm)	4.61a	0.39	4.64a	0.39	4.43b	0.43
Seed length (mm)	7.87a	0.55	7.81ab	0.40	7.64b	0.74
Seed moisture content (%)	7.00a	0.85	6.77ab	0.82	6.58b	0.87
Seed thickness (mm)	6.14a	0.22	6.10ab	0.20	6.06b	0.24
Seed width (mm)	6.09a	0.70	6.07a	0.49	6.05a	0.68
Total germination	7.34a	0.99	7.04ab	1.03	6.68b	1.14
Terminal leaf length (cm)	9.86a	0.77	9.94a	0.91	9.36b	0.81
Terminal leaf width (cm)	3.69a	0.33	3.77a	0.35	3.42b	0.36
Total seed weight (g)	66.93a	23.74	61.14ab	22.91	53.06b	18.77
100 seed weight (g)	19.60a	1.56	19.38a	1.64	19.21a	2.83
Qualitative (ordinal)						
Flower color	1.99a	0.205	1.95a	0.384	1.89a	0.319
Seed color	1.99a	1.64	1.95a	1.53	1.89a	1.65
Seed shape	1.95a	0.63	1.95a	0.7	1.89a	0.65

Qualitative (binary)	No. present	No. absent	Sig.	No. present	absent	Sig.	No. present	No. absent	Sig.
MASPIG	1	71	a	61	0	b	0	36	a
BRAPIG	0	72	a	61	0	b	0	36	a
PETPIG	0	72	a	61	0	b	0	36	a
GHABIT	30	42	a	34	27	ab	9	27	b
Seed variegation	22	50	a	33	38	b	2	34	c
Pod morphology	6	66	a	12	49	b	31	5	c
Pod shattering	18	54	a	15	46	a	1	35	b

SD, standard deviation; MASPIG, main stem pigmentation; BRAPIG, branch pigmentation; PETPIG, petiole pigmentation; GHABIT, growth habit; No. number; sig; significance level

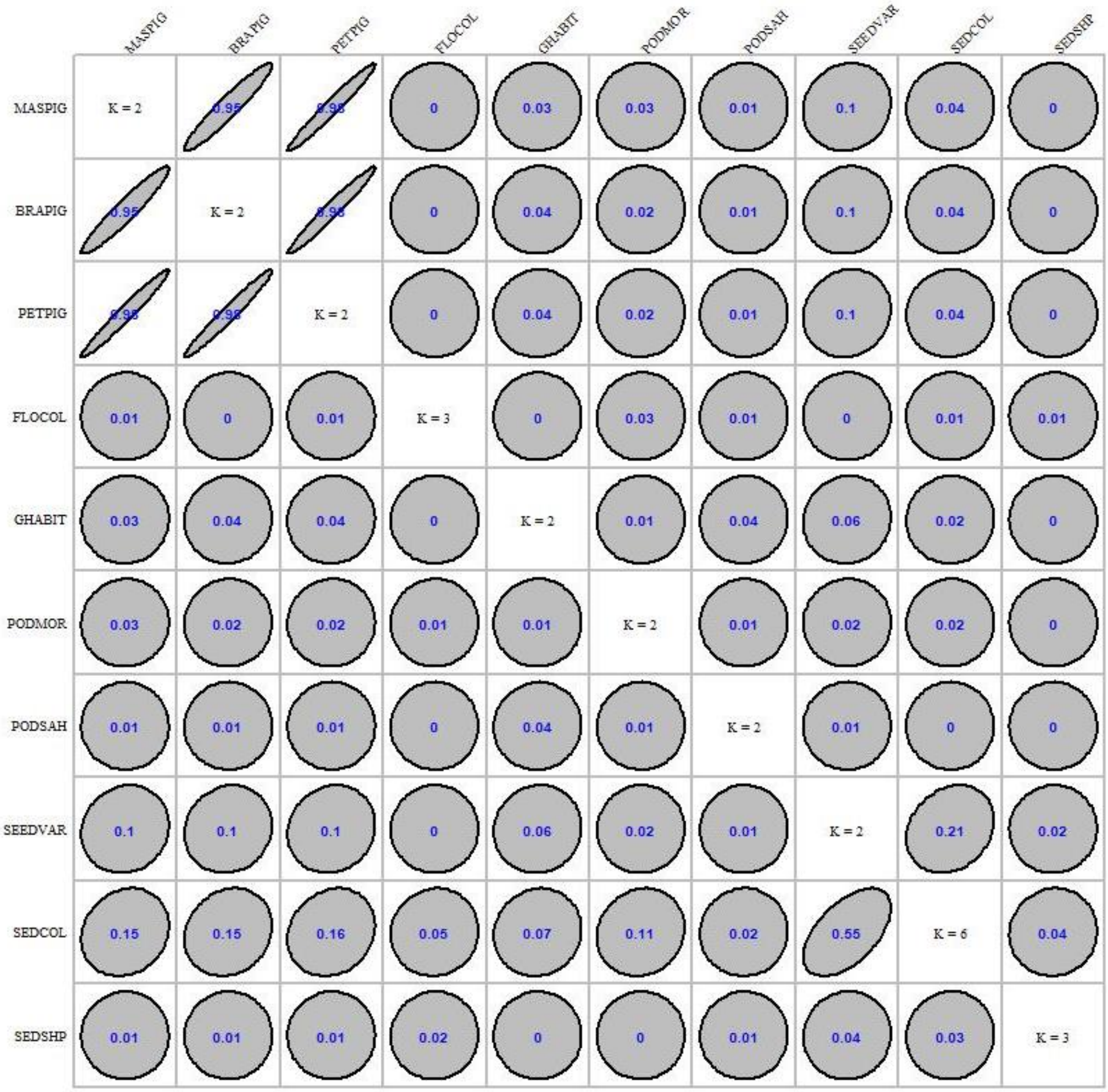

Supplementary Fig. S1: Correlation among 10 phenotypic traits (qualitative) evaluated across 169 AYB accessions. The traits were analyzed using the Goodman Kruskal package in R software version 4.1.1[56]. The x and y-axis represent the respective scales of each variable under consideration. The K values represent the levels at which data were collected for each trait. The forward association for each trait is shown above the diagonal, while the backward associations are shown below the diagonal. MASPIG, main stem pigmentation; BRAPIG, branch pigmentation; PETPIG, petiole pigmentation; FLOCOL; flower color; GHABIT, growth habit; PODMOR, pod morphology; PODSAH, pod shattering; SEDVAR, seed variegated; SEDCOL, seed color; SEDSHP, seed shape.

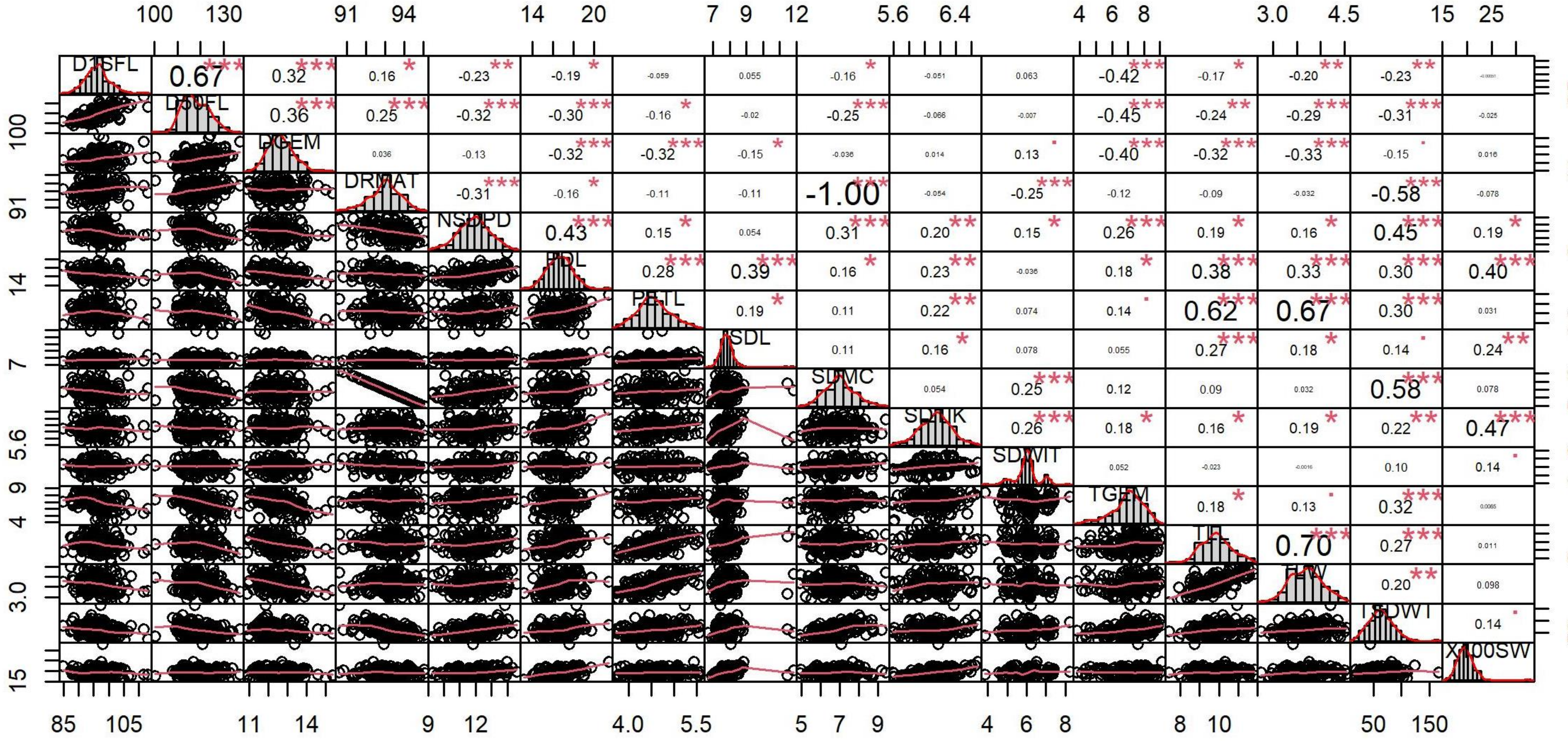

Supplementary Fig. S2: Correlation among 16 phenotypic traits (quantitative) evaluated across 169 AYB accessions. The associations of the quantitative traits were studied using Person's correlation package in R software version 4.1.1 [56]. The x and y-axis represent the respective scales of each variable under consideration. Below the diagonal are histograms showing the distribution of the traits. D1SFL, days to 1st flowering; D50FL, days to 50% flowering; DGEM, days to germination; DRMAT, dry seed matter; NSDPD, number of seed per pod; PDL, pod length; PETL, petiole length; SDL, seed length; SDMC, moisture content; SDTIK, seed thickness; TGEM, total germination; SDWIT, seed width; TLL, terminal leaf length; TLW, terminal leaf width; TSDWT, total seed weight; 100SW, 100 seed weight.

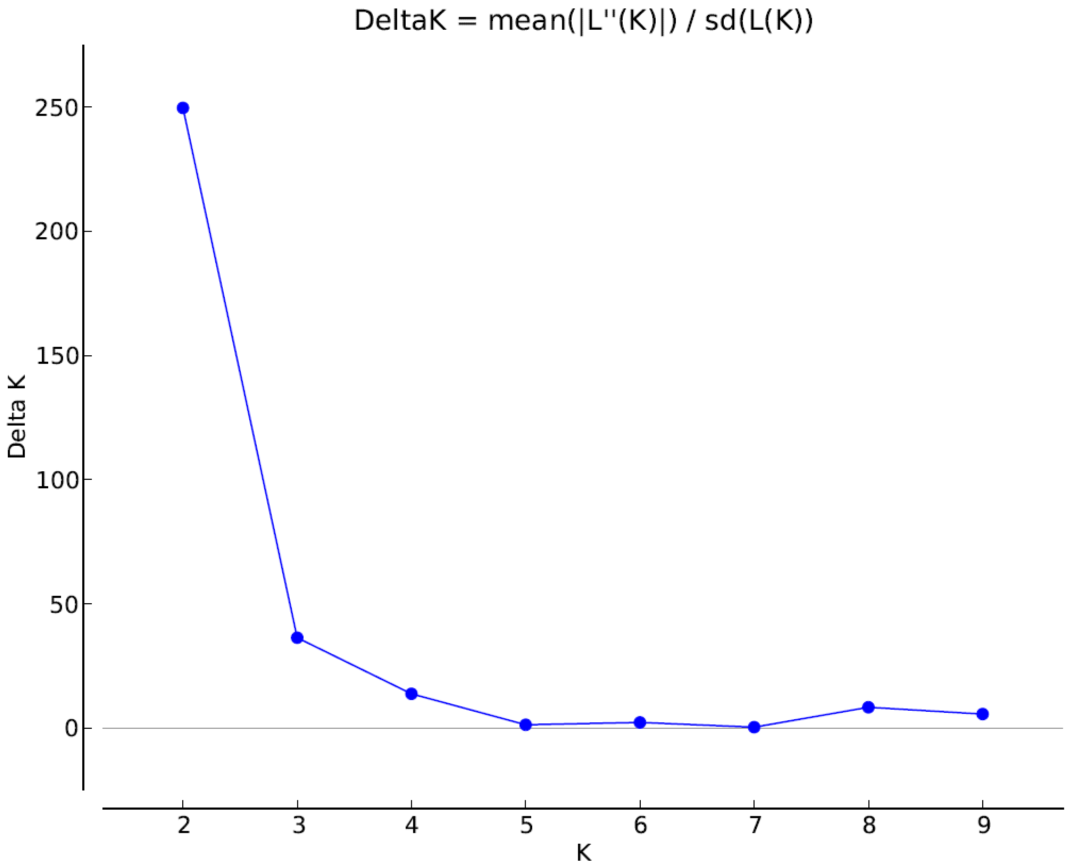

Supplementary Fig. S3: Optimal number of Delta K for different numbers of subpopulations. The Delta K plot was generated using STRUCTURE HARVESTER [60].

Supplementary Table S4: Percentage of variation explained by the first 3 PCoA axes

PCoA axis	1	2	3
% Variation	5.87	3.98	3.28
% Cumulative variation	5.87	9.84	13.12

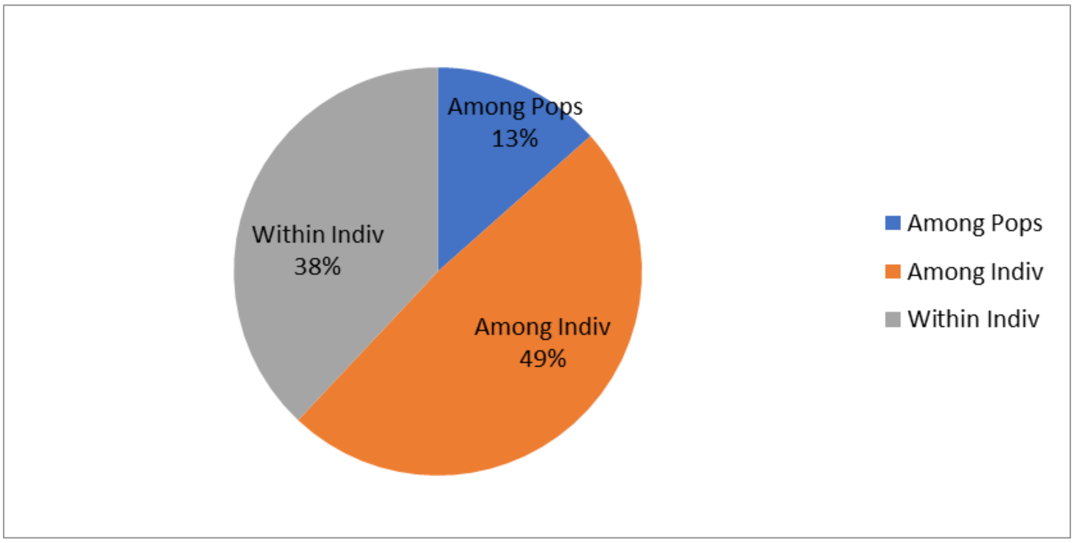

Supplementary Fig. S4. Percentages of Molecular Variance Among Population, Among Individuals, and Within Individuals. The figure was created in GenAlex software version 6.501 [61,62].

Supplementary Table S5. Monte-Carlo test for distant matrixes of genotypic data vs. phenotypic data, genotype data vs. combined data, and phenotypic data vs. combined data

Matrix	Parameters	Rxy	P-value	Alternative hypothesis
X vs Y	Genotypic matrix vs phenotypic matrix	0.02	0.24	greater
X vs Y	Genotypic matrix vs combined matrix	0.22	P < 0.01	greater
X vs Y	Phenotypic matrix vs combined matrix	0.96	P < 0.01	greater

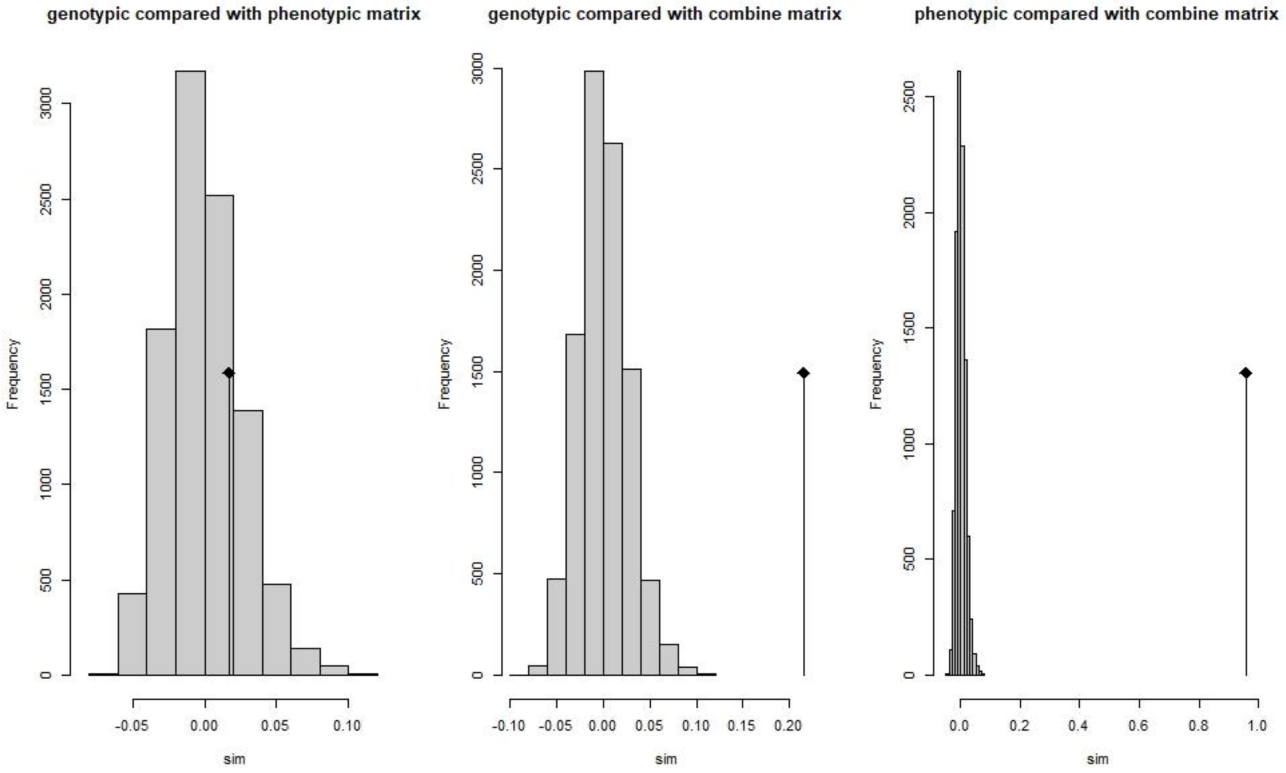

Supplementary Fig S5. Monte-Carlo correlation test between distant matrixes of genotypic data vs. phenotypic data, genotype data vs. combined data, and phenotypic data vs. combined data. Sim = similarity coefficient. The frequency plots were created using R software version 4.1.1[56].
